# Ovariotomy

**Published:** 1882-03

**Authors:** Theod. Billroth

**Affiliations:** Vienna


### Clinical Reports.

ARTICLE VI.

OVARIOTOMY. BY Professor THEOD. BILLROTH, Vienna. Reported by Dr. R. Stansbury Sutton, Formerly Lecturer on Gynæcology, Rush Medical College, Chicago.

This patient, Josepha Stedoma, is fifty-six years of age. She was married for eight years, during which time she gave birth to five children; the last was born in 1861. She is now a widow. Two years ago she observed a swelling in the abdomen. This swelling has constantly increased, and has at times been accompanied with pain. She has not complained of any distress in the functions of the bladder. During the last six weeks she has suffered a good deal of pain in the abdomen, which has been very sensitive, even to slight pressure. Eight days ago this pain subsided to a marked extent. At present, when recumbent, she has no pain, but when she walks she has some discomfort in the right hypochondriac region. The patient began to menstruate regularly at twenty years of age, and menstruation ceased at forty-eight. She is moderately large. She is anæmic, but her heart and lungs are normal. Dullness on percussion begins at the fifth rib on the right side, and extends over the enlarged abdomen. The abdomen is free from spots or marks upon the surface, and is also free from œdema and enlarged veins. The skin is tolerably tense.

| | | |
|---|---|---|
| Circumference at the umbilicus.... .......... | 39 in............ | 98 Cent. |
| From zyphoid cartilage to umbilicus.......... | 6 in............ | 15 " |
| From symphysis pubis........................ | 4¾ in............ | 12 " |
| From sup. spinous process of ilium to umbilicus on both sides, | 11 in. | 28 " |

The abdomen is moderately well distended, and the skin is tense. Fluctuation is distinct at all points. On palpation, there is found on the left side a peculiar parchment-like crepitation. Two fingers' breadth below the zyphoid, to the left side, the dullness begins, and extends downward to the symphysis pubis and laterally into the lumbar region. A vaginal examination reveals the os uteri high in the pelvis and the uterus movable.

OPERATION.

The abdomen was well washed, scrubbed and shaved, and finally rubbed off with a compress wet with five per cent. solution of carbolic acid. The ordinary mixture of chloroform 100 parts, ether 30, and alcohol 30 parts was administered. An incision was now made in the linea alba seven or eight centimeters in iength, ending four centimeters above the symphysis pubis. The exposed multilocular cyst was now emptied of six litres of dark, heavy fluid. The small adhesions, quite numerous, were separated with the fingers. Firmer ones were tied with carbolized silk sutures and burnt off with Paquelin's cautery.

The cysts were emptied sufficiently for the removal of the solid portions. The pedicle was found to be from the left ovary. The pedicle was long and not twisted. With a clamp a furrow was now made across the pedicle, and in this furrow the pedicle was tied in two sections with carbolized silk ligatures, cut short. The cyst was now cut away with the knife. The stump of the pedicle was now over a sponge divided again exterior to the ligatures with Paquelin's cautery. The "toilette de peritoneum" was carefully made, and the wound was now closed with a row of deep sutures of silver wire and lead buttons, and a row of superficial carbolized silk sutures. The wound was now dressed with iodoform gauze, Mackintosh cloth and a binder.

In the evening, ten hours after the operation, the temperature was 37° and pulse 92. The next morning the pulse fell below 90, and did not again rise above it. The temperature never exceeded 37 5–10°. The wound healed by first intention, the sutures were removed on the seventh day, and the patient was discharged on the seventeenth day after the operation.

